# Free amino acid contents of selected Ethiopian plant and fungi species: a search for alternative natural free amino acid sources for cosmeceutical applications

**DOI:** 10.1007/s00726-021-03008-5

**Published:** 2021-06-09

**Authors:** Birhanu Nigusse Kahsay, Jörg Ziegler, Peter Imming, Tsige Gebre-Mariam, Reinhard H. H. Neubert, Lucie Moeller

**Affiliations:** 1 Department of Pharmaceutics and Social Pharmacy, School of Pharmacy, College of Health Sciences, Addis Ababa University, P. O. Box 9086, Addis Ababa, Ethiopia; 2 Department of Molecular Signal Processing, Leibniz Institute of Plant Biochemistry, Weinberg 3, 06120 Halle (Saale), Germany; 3 Department of Medicinal Chemistry and Clinical Pharmacy, Martin Luther University Halle-Wittenberg, Kurt-Mothes-Str. 3, 06120 Halle (Saale), Germany; 4 Institute of Applied Dermatopharmacy, Martin Luther University Halle-Wittenberg, Weinbergweg 23, 06120 Halle (Saale), Germany; 5 Department Centre for Environmental Biotechnology, Helmholtz Centre for Environmental Research, Permoserstr. 15, 04318 Leipzig, Germany

**Keywords:** Free amino acids, Natural moisturizing factors, Cosmeceuticals, Ethiopian plants, Oyster mushroom

## Abstract

Free amino acids (FAAs), the major constituents of the natural moisturizing factor (NMF), are very important for maintaining the moisture balance of human skin and their deficiency results in dry skin conditions. There is a great interest in the identification and use of nature-based sources of these molecules for such cosmeceutical applications. The objective of the present study was, therefore, to investigate the FAA contents of selected Ethiopian plant and fungi species; and select the best sources so as to use them for the stated purpose. About 59 different plant species and oyster mushroom were included in the study and the concentrations of 27 FAAs were analyzed. Each sample was collected, lyophilized, extracted using aqueous solvent, derivatized with Fluorenylmethoxycarbonyl chloride (Fmoc-Cl) prior to solid-phase extraction and quantified using Liquid Chromatography Electrospray Ionization Tandem Mass Spectrometric (LC-ESI–MS/MS) system. All the 27 FAAs were detected in most of the samples. The dominant FAAs that are part of the NMF were found at sufficiently high concentration in the mushroom and some of the plants. This indicates that FAAs that could be included in the preparations for the management of dry skin condition can be obtained from a single natural resource and the use of these resources for the specified purpose have both economic and therapeutic advantage in addition to fulfilling customer needs.

**Supplementary Information:**

The online version contains supplementary material available at 10.1007/s00726-021-03008-5.

## Introduction

Amino acids, often called “the building blocks of life”, are primary metabolites which play a vital role in nutrition and health maintenance (Leuchtenberger et al. 2005; Mueller and Huebner 2003). They are used as ingredients in cosmetic and pharmaceutical products and as special nutrients in the medical field (Ikeda 2003; Park and Lee 2008). They are commonly employed in transfusions (Naylor et al. 1989; Louard et al. 1990; Stoimenova et al. 2013), in the manufacture of artificial sweeteners such as aspartame and as intermediate precursors for the production of antibiotics (Newsholme et al. 1985; Tang et al. 1994; Demain 2000; Garg et al. 2008). Amino acids, individually or in group, are also very important for the treatment of many disease conditions (Meletis and Barker 2005; Wu 2009; Lee and Kim 2019; Takaoka et al. 2019).

Free amino acids (FAAs) are also indispensable for healthy skin. They constitute the largest component (~ 40%) of the so-called natural moisturizing factor (NMF) (Jokura et al. 1995) and are very important in maintaining the moisture balance of skin. The most abundant FAAs within the NMF are l-serine (Ser) (~ 36%), l-glycine (Gly) (~ 22%) and l-alanine (Ala) (~ 13%) (Caspers et al. 2001). Citrulline (Cit), ornithine (Orn), histidine (His) and arginine (Arg) all account for 6–8%. In another study by Burke et al. (1966), Ser, Gly, Cit, Ala, His, and threonine (Thr), in that order, are the dominant FAAs in the horny layer of human skin and account for as much as 80%. Methionine (Met), cysteine (Cys), and tryptophan (Try) are present in smallest concentrations; and proline (Pro) is obscured because of the large amount of Cit masking its presence. Twenty-three (23) FAAs were detected in human corneocytes of 4 different study groups in a study by Hussain et al. (2019) at different concentrations. The level of NMFs including FAAs can decline in dry skin conditions due to many disease conditions such as atopic dermatitis, ichthyosis vulgaris, psoriasis, and age in addition to environmental conditions (Verdier-Sévrain and Bonté 2007; Kwan et al. 2012; Takada et al. 2012). The best way to overcome such disease condition seems to be the delivery of the major components of the NMF to the human skin in the form of moisturizers (Arezki et al. 2017). Even though much research work has not yet been done, FAAs have definitely a potential use in the cosmeceuticals.

FAAs for food, cosmeceutical and pharmaceutical applications can be obtained in four different methods, namely extraction (from natural resources), chemical synthesis, enzymatic synthesis, and fermentation (Leuchtenberger et al. 2005; Ikeda 2003). Despite the advancements in chemical synthesis and biotechnology, the need for herbal medicines is still at the top indicating the extraordinary relation between human beings and herbs, an almost mystical interdependence. As reported by the world health organization (WHO), about 80% of the total world population uses herbal medicines as their first-line primary health-care (Nagalingam 2017; Srivastava et al. 2019; Sharma et al. 2019). This resurgence of public interest in herbal remedies has been attributed to several factors including but not limited to the following: (a) herbal medicines are practically a mixture of many bioactive chemicals that can act synergistically (Sandberg and Corrigan 2001; Segneanu et al. 2017); (b) they might be more effective as compared to similar substances obtained through chemical synthesis (Thornfeldt 2005); (c) they are economically feasible and can be used by people at all economic levels; (d) they are preferable in terms of safety (side effects), contraindications and interactions with other substances (Segneanu et al. 2017); and (e) they have superior structural diversity, complex structure and multiple stereo centers (Phillipson 2001). There is also belief that herbal medicines are superior to manufactured products. In the cosmetic industry, herbal cosmeceuticals are the modern trend in the field of health, beauty and fashion; and there is a greater interest to use herbal materials. Herbal medicines are also highly believed to lead people to self-medication (Bandaranayake 2006). In addition to the reasons mentioned above, in cosmeceutical preparations that could contain the major constituents of the NMF (FAAs), use of a single natural source has a lot of economic advantage than using FAAs produced individually through any of the other options.

Understanding their benefits, the WHO has been working toward increasing the use of herbal medicines (WHO 2013) and currently about 25% of new drugs approved by Food and Drug Administration (FDA) and/or the European Medical Agency (EMA) are directly or indirectly plant based (Newman and Cragg 2012; Calixto 2019). Hence, research on plant-based bioactive molecules is among the top research topics in the pharmaceutical sector (Segneanu et al. 2017; Azmir et al. 2013; Chikezie et al. 2015). Due to such reasons, many countries have been extensively using their plants for pharmaceutical and cosmeceutical applications (Ji et al. 2017; Ruhsam and Hollingsworth 2018).

Ethiopia, a country endowed with a diverse biological resources and home of about 6500–7000 species of higher plants of which 12% are endemic, is one of the six regions rich in plant biodiversity (Berhan and Egziabher 2009) and these can be of course alternative sources of FAAs. Use of such sustainable and natural ingredients for cosmeceutical applications has a lot of benefits to the population in fostering sustainability and natural remedial approaches. Moreover, most of the major constituents of the NMF can be obtained from a single plant and this has superior advantage than the use of individual FAAs obtained through different options.

The objective of the present study was, therefore, to analyze the FAA contents of Ethiopian plants and mushroom and thus to identify those natural resources with highest amount of FAAs that can further be used for cosmeceutical applications in the management of dry skin conditions.

## Materials and methods

### Materials

A total of 60 plant and fungi species were collected from Gullele Botanical Garden and local supermarkets found in Addis Ababa, Ethiopia. All the plant and mushroom species were authenticated by the Ethiopian Biodiversity Institute, Addis Ababa, Ethiopia. Plant varieties such as cereals, leguminous plants, vegetables, fruits, spices and tea plants were included. In addition to the commonly used food items, attention was given to some indigenous aloe plants. One of the most common mushrooms found in Ethiopia, the *Pleurotus ostreatus* (Jacq. ex Fr.) P. Kumm., was also included in the study. Moreover, residuals from food processing such as the peals of fruits were also included. Prior consideration was given that the selected species can be cultivated and harvested at a rate conducive to production demands.

All the l-amino acid standards (Ala, Cys, Ser, Pro, His, Cit, Gly, Orn, Thr, Trp, Arg, Met, aspartate (Asp), glutamate (Glu), asparagine (Asn), glutamine (Gln), γ-aminobutyric acid (GABA), leucine (Leu), isoleucine (Ile), valine (Val), phenylalanine (Phe), lysine (Lys), *O*-acetylserine, oxyproline, methionine oxide, taurine (Tau), and tyrosine (Tyr)) and the internal standard, norvaline, were purchased from Sigma-Aldrich. Reagent grade *n*-pentane, sodium borate, and 9-fluorenylmethoxycarbonyl chloride (Fmoc-Cl) were also commercial products from Sigma-Aldrich. HPLC grade methanol and acetonitrile were used and these were purchased from Roth (Karlsruhe, Germany). Ultrapure water (resistivity 18.2 MΩ) purified by TKA X-CAD ultrapure water purification system (Thermo Fisher Scientific, Waltham, MA, USA) was used at all steps where water was required. Chromabond^®^ Multi 96 filter plates and Chromabond^®^ Sorbent HR-X were from Macherey–Nagel (Düren, Germany). All other reagents were of analytical grade.

### Methodology

The analysis of FAAs was conducted as per the method reported elsewhere (Ziegler et al. 2019). The procedure is briefly described in the following sections.

#### Sample extraction

Each sample was collected in triplicate and the collected samples were freeze dried (Alpha 2–4-LSC, Martin Christ Gefriertrocknungsanlagen GmbH, Germany). Five milligrams (5 mg) of each of the lyophilized samples was weighed using dual range analytical balance (Model XA105, Mettler Toledo, USA) and transferred to a 2 mL Eppendorf tube. A steal bead of 5 mm in diameter was inserted to each Eppendorf tube and the samples were pulverized in a mixer mill (Model MM 301, Retsch GmbH, Germany) at 25 s^−1^ for 50 s. Two hundred microliters (200 µL) of extraction solvent [a mixture of water and 10 mM norvaline (1 mL: 5 µL)] was added to each sample and the samples were mixed thoroughly for 20 min on a vortex (JK Janke and Kunkel IKA, model IKA VIBRAX-VXR). The samples were then centrifuged (Model 5415C, Eppendorf^®^, Germany) at 10,000×*g* for 5 min and the supernatant was transferred to a 1.5 mL Eppendorf tube. This solution was again centrifuged (Model 5415C, Eppendorf^®^, Germany) at 10,000×*g* for 5 min and the supernatant was transferred to a new 1.5 mL Eppendorf tube. This solution was stored in deep freezer at − 80 °C until the next process.

#### Standard preparation

Twenty millimolar (20 mM) stock solution of each amino acid standard was prepared in ultrapure water. Five microliters (5 μL) of each of the resulting solutions was transferred to a 2 mL Eppendorf tube and the resulting mixture was diluted to 500 μL with the same solvent. Finally, serial standard solutions were prepared for each amino acids and the internal standard to get a final concentration of 0, 2, 4, 8, 16, 64 and 128 pmol/µL after derivatization. These standard solutions were stored in deep freezer until the next step.

#### Sample derivatization and processing

After thawing at room temperature, 25 µL of the standard and sample solutions were transferred to 1.5 mL Eppendorf tubes. Fifty microliters (50 μL) of 0.5 M sodium borate buffer pH 7.9 and 100 μL 6 mM Fmoc-Cl solution (in acetone) were added to each solution and the resulting mixture was incubated for at least 5 min after mixing. Five hundred microliters (500 μL) of *n*-pentane was added to each solution, mixed thoroughly, centrifuged (Model 5415C, Eppendorf^®^, Germany) at 10,000×*g* for 1 min and the upper (organic phase) was discarded. This step was repeated two more times. After the last extraction step and removal of the organic phase, the tubes were opened and allowed to stand in a fume hood for evaporation of any residual organic solvent.

#### Solid-phase extraction

Solid-phase extraction (SPE) was conducted using Chromabond Multi 96-well plate (Macherey–Nagel, Düren, Germany) containing 50 mg/well HR-X-resin (Macherey–Nagel, Düren, Germany). First, the SPE plate was conditioned by 1 mL of methanol followed by 1 mL of water. In this and all subsequent steps, the liquid was passed through the resin by centrifugation at 500×*g* for 5 min using JS5.3 swingout rotor in an Avanti J-26XP centrifuge (Beckman Coulter, Fullerton, CA, USA). 500 µL of 5% (v/v) acetonitrile was added to the sample and standard solutions mentioned in “Sample derivatization and processing”. Then, the resulting solutions were quantitatively loaded onto the SPE plate, washed with 1 mL of water and the flow through was discarded after centrifugation. In the next step, 1 mL of methanol was added into the 96-deep well plate and eluted to a new block by centrifugation. Finally, the eluates were transferred from the 96-deep well block to 2 mL Eppendorf tubes, and allowed to evaporate under vacuum in an Eppendorf Concentrator (Model 5301, Eppendorf, Hamburg, Germany) at 45 °C for 45 min. Finally, the samples were centrifuged (Model 5415C, Eppendorf^®^, Germany) at 10,000×*g* for 10 min and the supernatant was transferred to the 96-well plate and the plate was placed in LC–MS/MS auto-sampler.

#### LC-ESI–MS/MS analysis

Chromatographic separation was achieved using Agilent 1290 liquid chromatography system equipped with Zorbax Eclipse Plus C18 Rapid Resolution HD column (2.1 × 50 mm, 1.8 µm, Agilent). The column temperature was maintained at 30 °C. Gradient elution with solvent A (0.2% v/v acetic acid in water) and solvent B (0.2% v/v acetic acid in acetonitrile) was used as mobile phase at a flow rate of 700 µL/min. Solvent A was held constant at 75% for 0.3 min and decreased to 50% over the next 6.7 min. Then, it was held at 2% over the next 0.7 min and increased to 75% for the next 0.4 min. Ten microliters (10 µL) and 4 µL were injected in to the auto-sampler for the sample and standard solutions, respectively.

Detection was done using API 3200 Triple Quadrupole LC–MS/MS system equipped with an ESI Turbo Ion Spray interface, operated in the negative ion mode (AB Sciex). The ion source parameters were set as follows: curtain gas was used at a pressure of 30 psi. The ion spray voltage was − 4500 V and the ion source temperature was set at 350 °C. Both the nebulizing and drying gas pressure were set at 50 psi. Triple quadrupole scans were acquired in the multiple reaction monitoring (MRM) mode with Q1 and Q3 set at “unit” resolution. Scheduled MRM was performed with a window of 90 s and a target scan time of 0.5 s. The mass spectrum (MS) parameters describing the MRMs for each FMOC-Cl derivatized amino acid were as reported by Ziegler et al. (2019).

#### Data analysis

The data analysis was done by automatic integration using Analyst software. A calibration curve was constructed using the standard solutions and from the graph the slope of the regression line was determined. The concentration (conc) of each FAA in nmol/mg was determined using the following equation and these results were converted to mg/g:

$${\text{Conc}}\left( {\frac{{{\text{nmol}}}}{{{\text{mg}}}}} \right) = \frac{{\left( {{\text{Peak}}\;{\text{area}}\;{\text{of}}\;{\text{analyte/Peak}}\;{\text{area}}\;{\text{of}}\;{\text{norvaline}}} \right) \times {\text{nmol}}\;{\text{of}}\;{\text{norvaline}}\;{\text{in}}\;{\text{the}}\;{\text{extraction}}\;{\text{solvent}}}}{{\left( {{\text{Slope}}\;{\text{of}}\;{\text{analyte/Slope}}\;{\text{of}}\;{\text{norvaline}}} \right) \times {\text{Sample}}\;{\text{weight}}\;{\text{(mg)}}}}$$

## Results and discussion

The FAA contents of the different plant and mushroom species included in the present study are shown in Table 1. Evidently, the concentrations are significantly different and the total FAAs found in the water extracts of the different species tested ranged from 0.86 mg/g (peal of mango, *Mangifera indica* L.) to 400.01 mg/g (oyster mushroom, *Pleurotus ostreatus* (Jacq. ex Fr.) P. Kumm.) as calculated on dry basis. All the tested 27 FAAs were found in most of the samples at varying concentrations (taurine (Tau), methionine oxide, *O*-acetylserine and oxyproline were analyzed but the results are not included in Table 1 as the concentrations were very low).

**Table 1:** Free amino acid content of different Ethiopian plant and mushroom species

S/n	Plants	Part	Concentration (mg/g)											
			Ala	Cys	Citr	Asp	Glu	Phe	Gly	GABA	His	Ilu	Lys	Leu
1	*Allium sativum* L. (garlic)	Tubers	11.48 ± 0.60	9.03 ± 0.01	5.53 ± 0.30	19.41 ± 0.62	23.3 ± 0.74	4.21 ± 0.49	1.93 ± 0.14	0.53 ± 0.02	6.6 ± 0.95	2.25 ± 0.11	18.93 ± 0.78	4.07 ± 0.30
2	*Allium spathaceum* Steud. ex A.Rich (Ethiopian onion)	Tubers	5.00 ± 0.51	6.17 ± 0.61	1.05 ± 0.08	13.79 ± 1.59	14.89 ± 1.25	5.69 ± 0.48	1.47 ± 0.21	0.91 ± 0.16	7.86 ± 0.54	4.86 ± 0.48	14.77 ± 1.30	14.06 ± 1.40
3	*Aloe ankoberensis* Gilbert and Sebsebe	Leaves	0.74 ± 0.09	5.53 ± 0.53	1.54 ± 0.18	3.05 ± 0.17	1.59 ± 0.13	0.99 ± 0.03	0.03 ± 0.00	0.16 ± 0.01	1.95 ± 0.07	0.38 ± 0.00	18.7 ± 0.02	0.49 ± 1.41
4	*Aloe benishangulana* Sebsebe & Tesfaye	Leaves	1.08 ± 0.05	0.46 ± 0.04	ND	0.53 ± 0.05	0.93 ± 0.05	0.60 ± 0.07	0.09 ± 0.01	0.88 ± 0.05	0.23 ± 0.03	0.56 ± 0.03	1.21 ± 0.11	0.78 ± 0.06
5	*Aloe debrana* Christian	Leaves	0.96 ± 0.15	0.40 ± 0.04	ND	0.28 ± 0.03	0.77 ± 0.07	0.33 ± 0.34	0.10 ± 0.01	0.65 ± 0.10	0.19 ± 0.01	0.44 ± 0.06	0.92 ± 0.19	0.57 ± 0.12
6	*Aloe percrassa* Tod	Leaves	0.61 ± 0.05	7.15 ± 1.74	1.05 ± 0.39	3.46 ± 1.05	1.34 ± 0.31	1.05 ± 0.03	0.04 ± 0.01	0.3 ± 0.13	2.07 ± 0.07	0.62 ± 0.00	20.82 ± 0.21	0.69 ± 0.43
7	*Aloe pirottae* Berger	Leaves	0.48 ± 0.07	3.93 ± 0.57	0.18 ± 0.04	1.75 ± 0.44	0.44 ± 0.10	0.8 ± 0.07	0.04 ± 0.01	0.17 ± 0.03	0.89 ± 0.11	0.37 ± 0.00	11.24 ± 0.07	0.54 ± 1.44
8	*Aloe sinana* Reynolds	Leaves	0.36 ± 0.09	4.33 ± 2.92	0.46 ± 0.29	2.81 ± 1.85	0.80 ± 0.54	0.7 ± 0.12	0.03 ± 0.01	0.22 ± 0.08	1.31 ± 0.30	0.34 ± 0.00	17.76 ± 0.10	0.58 ± 1.14
9	*Aloe tewoldei* Gilbert & Sebsebe	Leaves	0.35 ± 0.07	6.30 ± 0.71	1.11 ± 0.83	4.56 ± 0.54	1.38 ± 0.16	0.61 ± 0.11	0.02 ± 0.01	0.27 ± 0.08	1.94 ± 0.72	0.34 ± 0.00	19.18 ± 0.11	0.43 ± 1.35
10	*Ananas comosus* (L.) Merr. (pineapple)	Fruit	1.04 ± 0.46	1.12 ± 0.19	0.06 ± 0.02	4.64 ± 0.26	2.85 ± 0.19	0.83 ± 0.27	0.17 ± 0.00	1.07 ± 0.32	0.62 ± 0.15	0.38 ± 0.15	3.46 ± 0.84	0.85 ± 0.16
11	*Arachis hypogaea* L. (peanut)	Seeds	4.93 ± 0.02	0.86 ± 0.01	0.13 ± 0.00	2.36 ± 0.09	2.10 ± 0.23	7.07 ± 0.13	0.44 ± 0.01	3.05 ± 0.00	0.86 ± 0.02	1.34 ± 0.01	1.45 ± 0.05	3.38 ± 0.01
12	*Avena abyssinica* Hochst. (Ethiopian oat)	Seeds	0.14 ± 0.23	0.06 ± 0019	0.02 ± 0.06	0.54 ± 1.37	0.42 ± 0.97	0.04 ± 0.38	0.04 ± 0.09	0.05 ± 0.07	0.08 ± 0.74	0.05 ± 0.24	0.11 ± 0.11	0.06 ± 0.28
13	*Brassica carinata* A.Braun (Ethiopian mustard)	Seeds	9.13 ± 0.43	4.06 ± 0.58	0.1 ± 0.03	26.23 ± 0.64	26.4 ± 0.37	6.98 ± 0.48	2.22 ± 0.18	16.1 ± 0.86	1.68 ± 0.20	5.3 ± 0.37	3.76 ± 0.29	3.78 ± 0.43
14	*Brassica nigra* L. (black mustard)	Leaves	1.57 ± 0.12	6.24 ± 0.85	0.94 ± 0.09	15.3 ± 0.82	2.71 ± 0.01	2.02 ± 0.58	0.46 ± 0.02	1.68 ± 0.18	1.47 ± 0.71	8.66 ± 3.70	1.30 ± 0.19	8.93 ± 3.70
15	*Brassica oleracea* var *italica* (broccoli)	Leaves	11.79 ± 0.08	9.10 ± 0.42	0.27 ± 0.01	25.06 ± 1.94	21.01 ± 1.73	12.53 ± 0.10	4.61 ± 0.05	3.09 ± 0.05	18.03 ± 3.56	9.78 ± 0.12	8.40 ± 0.21	8.37 ± 0.20
16	*Brassica oleracea* var. *capitata* (cabbage)	Leaves	20.79 ± 1.74	8.60 ± 0.35	0.21 ± 0.03	22.89 ± 1.72	19.57 ± 0.05	3.19 ± 0.03	3.94 ± 0.60	5.85 ± 0.84	8.82 ± 0.41	7.62 ± 0.26	6.62 ± 0.02	8.27 ± 0.40
17	*Cannabis sativa* L. (hemp)	Leaves	2.51 ± 0.18	4.90 ± 0.65	0.73 ± 0.09	17.47 ± 1.7	9.82 ± 0.14	2.89 ± 0.12	0.40 ± 0.02	3.13 ± 0.10	2.13 ± 0.33	1.88 ± 0.32	8.77 ± 0.22	2.57 ± 0.14
18	*Capsicum annuum* L. (hot peppers)	Fruit	19.42 ± 0.64	5.02 ± 0.26	0.91 ± 0.08	10.25 ± 1.33	5.46 ± 0.80	3.76 ± 0.11	1.89 ± 0.11	14.33 ± 1.23	4.28 ± 0.91	4.00 ± 0.10	4.12 ± 0.81	10.86 ± 0.31
19	*Carica papaya* L. (papaya)	Fruit	3.73 ± 0.97	0.74 ± 0.33	0.78 ± 0.01	3.32 ± 0.32	2.17 ± 0.34	0.96 ± 0.19	1.77 ± 0.25	2.72 ± 0.32	0.91 ± 0.11	1.36 ± 0.06	1.08 ± 0.49	1.32 ± 0.69
20	*Cinnamomum verum* J.Presl (cinnamon)	Bark	0.65 ± 0.41	0.18 ± 0.14	0.08 ± 0.02	0.39 ± 0.25	0.3 ± 0.16	0.25 ± 0.21	0.25 ± 0.07	0.78 ± 0.58	0.65 ± 0.25	0.29 ± 0.19	0.69 ± 0.56	0.67 ± 0.52
21	*Citrullus lanatus* (Thunb.) Matsum. & Nakai (watermelon)	Seeds	7.28 ± 0.07	0.89 ± 0.04	0.39 ± 1.08	3.89 ± 0.20	4.39 ± 0.29	0.75 ± 0.03	0.51 ± 0.02	3.7 ± 0.10	1.19 ± 0.08	0.86 ± 0.03	1.39 ± 0.05	1.86 ± 0.05
22	*Citrullus lanatus* (Thunb.) Matsum. & Nakai (watermelon)	Fruit	0.10 ± 0.06	0.07 ± 0.01	1.04 ± 0.00	0.17 ± 0.03	0.23 ± 0.11	0.08 ± 0.00	0.05 ± 0.00	0.11 ± 0.04	0.15 ± 0.01	0.09 ± 0.00	0.24 ± 0.00	0.17 ± 0.00
23	*Coriandrum sativum* L. (coriander)	Leaves	0.85 ± 0.03	1.95 ± 0.49	1.92 ± 0.38	15.37 ± 3.00	1.19 ± 0.27	0.97 ± 0.13	0.11 ± 0.01	0.23 ± 0.02	0.79 ± 0.18	0.7 ± 0.00	4.94 ± 0.10	0.52 ± 2.48
24	*Cucumis sativus* L. (cucumber)	Fruit	1.32 ± 0.17	0.28 ± 0.06	0.23 ± 0.05	1.66 ± 0.15	1.38 ± 0.21	0.25 ± 0.02	0.18 ± 0.01	1.03 ± 0.07	0.22 ± 0.06	0.11 ± 0.02	0.40 ± 0.06	0.18 ± 0.03
25	*Cucurbita maxima* Duchesne (pumpkin)	Seeds	0.21 ± 0.07	0.42 ± 0.13	0.09 ± 0.06	2.26 ± 0.81	3.14 ± 0.91	0.39 ± 0.06	0.07 ± 0.02	0.21 ± 0.11	0.54 ± 0.14	0.12 ± 0.00	0.55 ± 0.10	0.13 ± 0.03
26	*Cucurbita pepo* subsp. *pepo* convar. giromontiina (zucchini)	Fruit	2.79 ± 0.43	0.96 ± 0.20	1.54 ± 0.05	10.54 ± 4.61	3.35 ± 1.55	0.87 ± 0.14	0.26 ± 0.06	3.68 ± 0.69	0.72 ± 0.11	0.46 ± 0.07	0.89 ± 0.19	0.46 ± 0.11
27	*Cuminum cyminum* L. (cumin)	Seeds	13.21 ± 0.54	4.40 ± 0.15	0.26 ± 0.02	17.45 ± 1.27	17.44 ± 1.64	5.71 ± 0.29	2.85 ± 0.19	4.69 ± 0.48	6.34 ± 0.24	4.42 ± 0.29	5.09 ± 0.01	4.85 ± 0.74
28	*Cymbopogon citratus* (DC.) Stapf (lemon grass)	Leaves	1.36 ± 0.41	1.97 ± 0.56	0.16 ± 0.09	3.13 ± 0.44	3.52 ± 1.06	1.52 ± 0.24	0.14 ± 0.06	0.74 ± 0.27	1.57 ± 0.61	0.36 ± 0.08	4.13 ± 01.67	0.64 ± 0.14
29	*Daucus carota* subsp. *sativus* (Hoffm.) Arcang. (carrot)	Tubers	9.33 ± 0.08	1.14 ± 0.46	0.46 ± 0.09	4.7 ± 0.72	5.36 ± 1.30	0.87 ± 0.13	0.58 ± 0.03	3.87 ± 0.39	1.08 ± 0.20	1.01 ± 0.10	1.14 ± 0.02	2.15 ± 0.34
30	*Eragrostis tef* (Zucc.) Trotter (teff)	Seeds	14.16 ± 0.71	4.30 ± 0.29	0.37 ± 0.01	20.5 ± 1.19	23.77 ± 1.14	4.43 ± 0.26	4.82 ± 0.29	1.04 ± 0.09	5.83 ± 0.24	3.93 ± 0.00	11.39 ± 0.27	4.11 ± 0.48
31	*Fragaria* × *ananassa* Duchesne (strawberry)	Fruit	1.01 ± 0.05	0.72 ± 0.06	0.06 ± 0.04	2.97 ± 0.07	1.69 ± 0.10	0.15 ± 0.01	0.08 ± 0.00	0.79 ± 0.04	0.54 ± 0.13	0.09 ± 0.02	0.30 ± 0.04	0.12 ± 0.01
32	*Glycine max* (L.) Merr. (soybean)	Seeds	5.04 ± 0.10	1.32 ± 0.16	0.25 ± 0.07	14.39 ± 1.10	17.31 ± 2.74	2.81 ± 0.38	1.86 ±	2.66 ± 0.65	8.54 ± 0.60	2.01 ± 0.43	8.65 ± 0.18	3.40 ± 0.47
33	*Helianthus annuus* L. (sunflower)	Seeds	6.36 ± 0.49	3.52 ± 0.04	0.19 ± 0.07	14.34 ± 0.35	9.12 ± 0.39	8.04 ± 0.99	2.75 ± 0.51	0.70 ± 0.10	5.05 ± 0.76	6.52 ± 0.48	5.62 ± 0.80	5.81 ± 0.89
34	*Lactuca sativa* L. (lettuce)	Leaves	0.48 ± 0.03	5.85 ± 2.07	0.24 ± 0.08	13.00 ± 7.53	1.29 ± 0.61	0.48 ± 0.19	0.04 ± 0.01	0.25 ± 0.08	0.71 ± 0.10	0.48 ± 0.00	7.87 ± 0.16	0.59 ± 1.56
35	*Linum usitatissimum* L. (flax)	Seeds	9.92 ± 0.29	3.77 ± 0.20	0.08 ± 0.02	21.47 ± 0.78	14.61 ± 1.19	8.41 ± 0.64	2.92 ± 0.08	0.33 ± 0.09	4.28 ± 0.33	5.68 ± 0.30	9.78 ± 0.57	7.14 ± 1.63
36	*Malus domestica* Borkh. (apple)	Fruit	0.15 ± 0.01	0.03 ± 0.00	ND	0.56 ± 0.09	0.26 ± 0.02	0.01 ± 0.00	0.01 ± 0.00	0.12 ± 0.02	0.02 ± 0.00	0.03 ± 0.00	0.03 ± 0.00	0.02 ± 0.00
37	*Mangifera indica* L. (mango)	Fruit	0.30 ± 0.04	0.04 ± 0.00	0.02 ± 0.01	0.33 ± 0.01	0.23 ± 0.00	0.01 ± 0.00	0.01 ± 0.00	0.13 ± 0.02	0.03 ± 0.01	0.02 ± 0.00	0.05 ± 0.00	0.02 ± 0.00
38	*Mangifera indica* L. (mango)	Peal	0.09 ± 0.00	0.02 ± 0.02	0.01 ± 0.01	0.11 ± 0.00	0.07 ± 0.01	0.01 ± 0.01	0.01 ± 0.00	0.06 ± 0.01	0.02 ± 0.04	0.01 ± 0.01	0.02 ± 0.00	0.01 ± 0.02
39	*Matricaria chamomilla* L. (chamomile)	Flower	2.26 ± 0.13	4.78 ± 0.21	0.46 ± 0.01	7.62 ± 1.15	7.84 ± 1.05	2.81 ± 0.23	0.52 ± 0.09	1.60 ± 0.07	3.86 ± 0.62	2.09 ± 0.24	7.25 ± 0.05	2.31 ± 0.08
40	*Mentha* × *piperita* L. (peppermint)	Leaves	1.00 ± 0.07	1.11 ± 0.07	0.02 ± 0.01	2.69 ± 0.11	0.19 ± 0.02	2.08 ± 0.12	0.20 ± 0.01	0.72 ± 0.04	1.12 ± 0.09	0.37 ± 0.00	0.83 ± 0.04	0.27 ± 0.14
41	*Moringa oleifera* Lam. (moringa)	Leaves	17.73 ± 0.71	14.53 ± 0.71	0.71 ± 0.11	21.91 ± 0.04	18.03 ± 1.07	14.93 ± 1.44	3.20 ± 0.36	10.58 ± 1.15	7.23 ± 0.76	12.5 ± 0.84	13.1 ± 0.38	13.29 ± 1.14
42	*Musa acuminata* Colla (banana)	Peal	0.11 ± 0.01	0.13 ± 0.01	ND	0.50 ± 0.05	0.23 ± 0.05	0.01 ± 0.00	0.05 ± 0.00	0.29 ± 0.03	0.73 ± 0.18	0.03 ± 0.00	0.12 ± 0.03	0.17 ± 0.01
43	*Musa acuminata* Colla (banana)	Fruit	0.37 ± 0.06	0.18 ± 0.01	ND	0.54 ± 0.03	0.41 ± 0.11	0.03 ± 0.00	0.05 ± 0.00	0.20 ± 0.04	0.08 ± 0.01	0.03 ± 0.00	0.08 ± 0.00	0.08 ± 0.00
44	*Nigella sativa* L. (black cumin)	Seeds	6.66 ± 0.49	2.41 ± 01	0.17 ± 0.01	9.63 ± 0.62	20.02 ± 0.28	7.51 ± 0.91	1.67 ± 0.09	1.78 ± 0.21	2.36 ± 0.16	2.97 ± 0.12	5.28 ± 0.20	3.79 ± 0.79
45	*Ocimum basilicum* L. (Mexican spice basil)	Leaves	1.59 ± 0.13	1.77 ± 0.12	0.26 ± 0.05	2.41 ± 0.29	0.27 ± 0.01	1.21 ± 0.11	0.20 ± 0.02	1.23 ± 0.08	1.02 ± 0.02	0.54 ± 0.00	2.8 ± 0.03	1.13 ± 0.15
46	*Persea americana* Mill. (avocado)	Fruit	6.94 ± 0.08	1.68 ± 0.08	0.14 ± 0.00	1.81 ± 0.08	2.44 ± 0.32	1.01 ± 0.08	1.38 ± 0.04	20.3 ± 1.07	0.64 ± 0.25	2.09 ± 0.14	3.69 ± 1.01	3.61 ± 0.93
47	*Phaseolus vulgaris* L. (common bean)	Seeds	16.39 ± 0.06	4.46 ± 0.01	0.79 ± 0.01	9.98 ± 0.17	4.56 ± 0.14	3.49 ± 0.04	1.65 ± 0.02	12.48 ± 0.01	3.58 ± 0.22	3.72 ± 0.01	4.2 ± 0.03	7.95 ± 0.03
48	*Piper nigrum* L. (black pepper)	Fruit	6.74 ± 0.48	1.29 ± 0.04	0.10 ± 0.04	54.06 ± 4.26	2.16 ± 0.35	0.89 ± 0.09	0.94 ± 0.11	10.5 ± 0.82	0.93 ± 0.02	0.96 ± 0.02	1.68 ± 0.01	1.16 ± 0.31
49	*Pisum sativum* var. *Abyssinicum* (A. Braun) Berger (decoco)	Seeds	5.79 ± 0.45	6.51 ± 0.07	0.72 ± 0.05	29.74 ± 2.38	28.38 ± 1.23	5.09 ± 0.29	2.79 ± 0.04	3.96 ± 0.46	3.59 ± 0.31	2.88 ± 0.47	9.15 ± 1.16	4.88 ± 0.23
50	*Plectranthus edulis* (Vatke) Angew (Ethiopian potato)	Tubers	5.45 ± 0.98	4.26 ± 0.53	0.18 ± 0.03	10.87 ± 0.26	3.31 ± 0.63	8.10 ± 0.86	1.35 ± 0.19	8.88 ± 1.42	2.53 ± 0.26	5.55 ± 0.73	7.13 ± 1.17	4.27 ± 0.66
51	*Pleurotus ostreatus* (Jacq. ex Fr.) P. Kumm. (oyster mushroom)	Fruit body	18.47 ± 0.76	23.42 ± 0.38	0.84 ± 0.13	20.62 ± 9.39	35.65 ± 6.26	19.6 ± 0.12	4.27 ± 0.31	1.38 ± 0.02	26.38 ± 2.36	13.39 ± 0.21	26.19 ± 02.78	17.13 ± 0.15
52	*Ruta chalepensis* L. (rue)	Leaves	12.71 ± 0.36	7.45 ± 0.46	0.25 ± 0.02	10.73 ± 0.71	4.64 ± 0.40	9.01 ± 0.62	2.41 ± 0.04	6.1 ± 0.26	4.64 ± 0.16	6.45 ± 0.35	8.10 ± 0.39	7.51 ± 0.15
53	*Rumex abyssinicus* Jacq. (mekmako)	Leaves	1.11 ± 0.15	1.83 ± 0.13	0.07 ± 0.02	2.40 ± 0.26	4.13 ± 0.58	1.29 ± 0.11	0.24 ± 0.03	5.8 ± 0.27	2.35 ± 0.22	1.71 ± 0.16	2.04 ± 0.12	1.43 ± 0.11
54	*Salvia rosmarinus* Schleid. (rosemary)	Leaves	4.63 ± 0.29	0.83 ± 0.06	0.12 ± 0.04	2.84 ± 0.25	2.42 ± 0.26	7.9 ± 0.25	0.52 ± 0.07	3.49 ± 0.59	1.11 ± 0.35	1.52 ± 0.22	1.78 ± 0.23	4.21 ± 0.37
55	*Sesamum indicum* L. (sesame)	Seeds	3.61 ± 0.02	1.06 ± 0.01	0.35 ± 0.01	1.88 ± 0.06	2.98 ± 0.07	1.19 ± 0.02	0.47 ± 0.00	4.34 ± 0.01	1.18 ± 0.04	1.04 ± 0.01	2.37 ± 0.02	2.65 ± 0.03
56	*Solanum lycopersicum* L. (tomato)	Fruit	2.77 ± 0.16	1.61 ± 0.49	0.51 ± 0.04	26.18 ± 3.68	25.65 ± 3.46	4.46 ± 0.55	0.24 ± 0.03	5.74 ± 0.86	4.62 ± 0.44	1.02 ± 0.21	3.97 ± 0.59	1.11 ± 0.17
57	*Syzygium aromaticum* (L.) Merr. & L.M.Perry (clove)	Flower	15.67 ± 0.01	6.04 ± 0.00	0.45 ± 0.00	8.07 ± 0.02	13.21 ± 0.30	12.77 ± 0.02	3.00 ± 0.00	32.88 ± 0.03	8.87 ± 0.01	10.23 ± 0.01	21.01 ± 0.01	25.71 ± 0.05
58	*Thymus vulgaris* L. (thyme)	Leaves	7.79 ± 0.67	2.90 ± 0.31	3.65 ± 0.19	5.50 ± 0.42	4.91 ± 0.45	2.43 ± 0.33	1.08 ± 0.41	4.57 ± 0.38	1.36 ± 0.53	2.61 ± 0.25	2.85 ± 0.55	3.83 ± 0.46
59	*Trigonella foenum-graecum* L. (fenugreek)	Seeds	6.29 ± 0.06	1.69 ± 0.04	0.21 ± 0.02	1.79 ± 0.06	2.56 ± 0.30	0.96 ± 0.11	1.25 ± 0.03	17.97 ± 0.04	0.79 ± 0.14	2.02 ± 0.03	4.02 ± 0.13	3.95 ± 0.24
60	*Zingiber officinale* Roscoe (ginger)	Tubers	20.77 ± 1.46	4.74 ± 0.00	1.01 ± 0.03	83.40 ± 0.13	20.65 ± 0.80	2.92 ± 0.34	2.83 ± 0.16	9.81 ± 0.29	3.21 ± 0.10	3.7 ± 0.12	6.59 ± 0.11	5.81 ± 0.16
Min			0.09	0.02	0.00	0.11	0.07	0.00	0.01	0.05	0.02	0.00	0.02	0.01
Max			20.79	23.42	5.53	83.4	35.65	19.6	4.82	32.88	26.38	6.26	26.19	25.71

S/n	Plants	Concentration (mg/g)												Total FAA
		Part analyzed	Met	Asn	Orn	Pro	Gln	Arg	Ser	Thr	Val	Try	Tyr	
1	*Allium sativum* L. (garlic)	Tubers	1.31 ± 0.22	25.31 ± 2.17	4.21 ± 0.34	3.56 ± 0.17	14.66 ± 1.42	16.42 ± 1.33	9.90 ± 0.8	7.60 ± 0.69	5.39 ± 0.29	3.96 ± 0.50	11.00 ± 1.74	225.09
2	*Allium spathaceum* Steud. ex A.Rich (Ethiopian onion)	Tubers	1.76 ± 0.78	24.84 ± 0.34	2.13 ± 0.18	2.14 ± 0.33	10.27 ± 0.60	9.98 ± 0.97	6.02 ± 0.58	5.42 ± 0.64	5.89 ± 0.69	10.26 ± 0.92	22.42 ± 3.24	188.01
3	*Aloe ankoberensis* Gilbert and Sebsebe	Leaves	0.01 ± 0.10	6.20 ± 0.01	0.35 ± 0.01	0.16 ± 0.00	12.44 ± 0.01	4.01 ± 0.11	2.42 ± 0.07	5.29 ± 0.21	0.45 ± 0.00	0.50 ± 0.03	6.35 ± 0.01	73.06
4	*Aloe benishangulana* Sebsebe & Tesfaye	Leaves	0.02 ± 0.01	0.52 ± 0.01	0.02 ± 0.00	0.50 ± 0.04	1.47 ± 0.10	0.58 ± 0.02	0.35 ± 0.01	0.44 ± 0.03	0.63 ± 0.04	0.20 ± 0.02	0.71 ± 0.05	12.28
5	*Aloe debrana* Christian	Leaves	0.01 ± 0.01	0.75 ± 0.29	0.09 ± 0.02	0.35 ± 0.03	0.40 ± 0.13	0.43 ± 0.10	0.26 ± 0.06	0.38 ± 0.06	0.50 ± 0.07	0.16 ± 0.04	0.72 ± 0.10	9.27
6	*Aloe percrassa* Tod	Leaves	0.01 ± 0.15	7.58 ± 0.24	0.38 ± 0.04	0.16 ± 0.00	10.21 ± 0.02	3.56 ± 1.16	2.15 ± 0.7	6.26 ± 0.30	0.52 ± 0.00	0.54 ± 0.14	5.68 ± 0.02	75.91
7	*Aloe pirottae* Berger	Leaves	0.00 ± 0.05	4.68 ± 0.11	0.23 ± 0.02	0.11 ± 0.00	3.85 ± 0.01	2.01 ± 0.14	1.21 ± 0.08	3.72 ± 0.12	0.34 ± 0.00	0.52 ± 0.05	2.16 ± 0.06	39.40
8	*Aloe sinana* Reynolds	Leaves	0.00 ± 0.24	7.01 ± 0.30	0.54 ± 0.07	0.12 ± 0.00	5.92 ± 0.05	3.19 ± 1.26	1.93 ± 0.76	3.94 ± 1.23	0.35 ± 0.01	0.31 ± 0.17	3.64 ± 0.07	56.86
9	*Aloe tewoldei* Gilbert & Sebsebe	Leaves	0.00 ± 0.01	6.10 ± 0.05	0.47 ± 0.10	0.13 ± 0.00	15.3 ± 0.05	3.80 ± 0.75	2.29 ± 0.46	6.14 ± 0.46	0.36 ± 0.00	0.34 ± 0.12	5.45 ± 0.09	76.70
10	*Ananas comosus* (L.) Merr. (pineapple)	Fruit	0.19 ± 0.07	20.14 ± 6.48	0.12 ± 0.01	0.28 ± 0.09	5.04 ± 3.65	4.55 ± 0.98	2.74 ± 0.59	1.30 ± 0.42	0.54 ± 0.14	0.15 ± 0.03	2.72 ± 0.50	54.68
11	*Arachis hypogaea* L. (peanut)	Seeds	0.03 ± 0.01	26.75 ± 0.18	0.48 ± 0.00	0.60 ± 0.02	4.80 ± 0.02	1.89 ± 0.02	1.14 ± 0.01	0.84 ± 0.01	1.65 ± 0.01	0.74 ± 0.01	7.84 ± 0.01	73.62
12	*Avena abyssinica* Hochst. (Ethiopian oat)	Seeds	0.00 ± 0.04	0.80 ± 2.81	0.01 ± 0.13	0.09 ± 1.09	0.26 ± 2.96	0.11 ± 0.58	0.07 ± 0.35	0.04 ± 0.28	0.05 ± 0.44	0.25 ± 0.76	0.22 ± 0.69	4.41
13	*Brassica carinata* A.Braun (Ethiopian mustard)	Seeds	2.00 ± 0.03	22.67 ± 2.12	0.11 ± 0.01	2.79 ± 0.45	2.01 ± 0.22	7.83 ± 0.42	4.72 ± 0.25	4.29 ± 0.12	4.86 ± 0.79	4.69 ± 0.16	4.26 ± 0.47	161.05
14	*Brassica nigra* L. (black mustard)	Leaves	1.75 ± 0.39	0.77 ± 0.43	1.33 ± 0.71	0.01 ± 0.00	0.53 ± 0.08	5.28 ± 1.64	3.19 ± 0.99	12.69 ± 0.03	0.03 ± 0.01	2.93 ± 0.44	0.70 ± 0.06	80.35
15	*Brassica oleracea* var *italica* (broccoli)	Leaves	5.20 ± 0.68	25.53 ± 0.27	2.46 ± 0.44	20.58 ± 0.36	27.01 ± 0.94	14.35 ± 0.3	8.65 ± 0.18	7.59 ± 0.25	12.25 ± 0.27	7.42 ± 0.13	21.86 ± 1.92	291.90
16	*Brassica oleracea* var. *capitata* (cabbage)	Leaves	0.92 ± 0.32	23.08 ± 0.31	0.60 ± 0.03	19.4 ± 2.89	32.10 ± 1.47	20.5 ± 2.57	12.37 ± 01.55	7.75 ± 0.88	10.16 ± 1.28	1.89 ± 0.43	11.24 ± 1.07	248.81
17	*Cannabis sativa* L. (hemp)	Leaves	0.04 ± 0.01	9.12 ± 1.11	0.30 ± 0.01	0.84 ± 0.10	11.03 ± 1.67	4.46 ± 0.55	2.69 ± 0.33	4.75 ± 0.66	2.44 ± 0.08	1.53 ± 0.13	9.64 ± 0.85	102.33
18	*Capsicum annuum* L. (hot peppers)	Fruit	0.12 ± 0.09	30.38 ± 3.08	0.98 ± 0.24	2.92 ± 0.08	27.22 ± 1.7	13.37 ± 0.02	8.07 ± 0.01	5.59 ± 0.52	4.79 ± 0.06	2.11 ± 0.03	4.06 ± 0.81	205.68
19	*Carica papaya* L. (papaya)	Fruit	0.10 ± 0.05	5.50 ± 0.23	1.10 ± 0.43	1.80 ± 0.33	0.35 ± 0.16	3.71 ± 0.01	2.24 ± 0.00	0.87 ± 0.01	0.77 ± 0.002	0.32 ± 0.09	1.18 ± 0.49	37.77
20	*Cinnamomum verum* J.Presl (cinnamon)	Bark	ND	2.14 ± 2.47	0.46 ± 0.08	0.17 ± 0.07	1.00 ± 1.09	1.11 ± 0.48	0.67 ± 0.29	0.24 ± 0.15	0.16 ± 0.14	0.12 ± 0.10	1.02 ± 0.57	12.12
21	*Citrullus lanatus* (Thunb.) Matsum. & Nakai (watermelon)	Seeds	0.03 ± 0.00	8.49 ± 0.03	0.67 ± 0.04	0.57 ± 0.06	6.43 ± 0.27	2.54 ± 0.05	1.53 ± 0.03	0.88 ± 0.04	1.02 ± 0.02	0.14 ± 0.01	1.38 ± 0.06	50.21
22	*Citrullus lanatus* (Thunb.) Matsum. & Nakai (watermelon)	Fruit	0.01 ± 0.00	0.26 ± 0.37	0.03 ± 0.00	0.07 ± 0.00	0.52 ± 0.06	0.26 ± 0.04	0.16 ± 0.03	0.07 ± 0.02	0.08 ± 0.00	0.05 ± 0.02	0.19 ± 0.00	4.20
23	*Coriandrum sativum* L. (coriander)	Leaves	0.01 ± 0.14	0.90 ± 0.07	0.35 ± 0.02	0.12 ± 0.00	1.58 ± 0.02	2.34 ± 0.37	1.41 ± 0.22	3.62 ± 0.27	0.89 ± 0.00	0.46 ± 0.14	1.94 ± 0.08	42.70
24	*Cucumis sativus* L. (cucumber)	Fruit	ND	0.50 ± 0.33	0.09 ± 0.01	0.13 ± 0.02	0.83 ± 0.52	0.99 ± 0.07	0.60 ± 0.04	0.30 ± 0.05	0.13 ± 0.02	0.12 ± 0.03	0.64 ± 0.03	11.67
25	*Cucurbita maxima* Duchesne (pumpkin)	Seeds	0.01 ± 0.01	1.19 ± 0.45	0.11 ± 0.03	0.06 ± 0.02	1.39 ± 0.66	0.58 ± 0.22	0.35 ± 0.13	0.24 ± 0.05	0.12 ± 0.02	1.76 ± 0.5	1.38 ± 0.62	16.11
26	*Cucurbita pepo* subsp. *pepo* convar. giromontiina (zucchini)	Fruit	ND	7.96 ± 1.00	0.12 ± 0.03	0.24 ± 0.06	11.78 ± 2.21	5.62 ± 0.87	3.39 ± 0.52	0.85 ± 0.15	0.58 ± 0.08	0.17 ± 0.04	1.56 ± 0.33	59.89
27	*Cuminum cyminum* L. (cumin)	Seeds	0.46 ± 0.07	37.30 ± 3.36	0.51 ± 0.13	13.38 ± 0.42	4.99 ± 0.05	5.08 ± 0.27	3.06 ± 0.16	4.03 ± 0.44	4.88 ± 0.65	2.80 ± 0.21	3.80 ± 0.06	164.20
28	*Cymbopogon citratus* (DC.) Stapf (lemon grass)	Leaves	0.08 ± 0.10	20.47 ± 6.35	0.15 ± 0.02	0.29 ± 0.07	6.84 ± 5.34	3.46 ± 0.73	2.09 ± 0.44	1.96 ± 0.53	0.71 ± 0.13	0.33 ± 0.02	5.47 ± 1.05	61.76
29	*Daucus carota* subsp. *sativus* (Hoffm.) Arcang. (carrot)	Tubers	0.03 ± 0.02	10.43 ± 1.37	0.79 ± 0.06	0.66 ± 0.09	8.10 ± 0.11	2.83 ± 0.21	1.71 ± 0.13	1.07 ± 0.14	1.24 ± 0.11	0.17 ± 0.07	1.24 ± 0.17	60.25
30	*Eragrostis tef* (Zucc.) Trotter (teff)	Seeds	1.22 ± 0.32	26.61 ± 0.25	0.51 ± 0.03	3.38 ± 0.02	4.38 ± 0.23	8.31 ± 0.59	5.01 ± 0.36	4.42 ± 0.2	4.08 ± 0.00	3.23 ± 0.44	5.81 ± 0.22	162.84
31	*Fragaria* × *ananassa* Duchesne (strawberry)	Fruit	ND	27.51 ± 0.97	0.08 ± 0.01	0.09 ± 0.01	0.67 ± 0.05	1.75 ± 0.11	1.05 ± 0.07	0.65 ± 0.02	0.16 ± 0.01	0.07 ± 0.01	0.63 ± 0.10	41.20
32	*Glycine max* (L.) Merr. (soybean)	Seeds	0.38 ± 0.07	27.48 ± 1.65	0.62 ± 0.06	1.73 ± 0.49	1.11 ± 0.01	3.52 ± 1.43	2.12 ± 0.87	1.71 ± 0.41	1.99 ± 0.41	7.45 ± 0.68	5.82 ± 1.63	130.91
33	*Helianthus annuus* L. (sunflower)	Seeds	2.48 ± 0.15	21.90 ± 0.90	0.27 ± 0.05	16.05 ± 0.77	4.52 ± 0.56	5.44 ± 0.89	3.28 ± 0.54	2.91 ± 0.18	4.75 ± 0.55	4.24 ± 0.54	6.28 ± 0.44	134.01
34	*Lactuca sativa* L. (lettuce)	Leaves	ND	24.94 ± 0.26	0.37 ± 0.03	0.08 ± 0.00	10.62 ± 0.01	4.54 ± 0.79	2.74 ± 0.48	5.57 ± 1.01	0.59 ± 0.00	0.43 ± 0.35	2.25 ± 0.02	83.51
35	*Linum usitatissimum* L. (flax)	Seeds	2.39 ± 0.20	20.03 ± 0.43	0.31 ± 0.04	5.28 ± 0.06	4.78 ± 0.48	5.82 ± 0.10	3.51 ± 0.06	3.82 ± 0.18	4.77 ± 0.40	6.62 ± 0.48	7.44 ± 0.48	148.71
36	*Malus domestica* Borkh. (apple)	Fruit	ND	0.43 ± 0.00	0.01 ± 0.00	0.02 ± 0.00	0.05 ± 0.01	0.11 ± 0.01	0.07 ± 0.00	0.03 ± 0.00	0.04 ± 0.00	ND	0.02 ± 0.00	2.01
37	*Mangifera indica* L. (mango)	Fruit	ND	0.06 ± 0.01	0.01 ± 0.00	0.05 ± 0.01	0.10 ± 0.00	0.16 ± 0.01	0.10 ± 0.01	0.03 ± 0.00	0.01 ± 0.00	ND	0.02 ± 0.00	1.72
38	*Mangifera indica* L. (mango)	Peal	ND	0.20 ± 0.01	0.01 ± 0.01	0.01 ± 0.00	0.05 ± 0.00	0.07 ± 0.02	0.04 ± 0.01	0.01 ± 0.01	0.01 ± 0.01	0.02 ± 0.02	0.01 ± 0.05	0.86
39	*Matricaria chamomilla* L. (chamomile)	Flower	0.06 ± 0.02	21.44 ± 0.61	0.23 ± 0.01	5.23 ± 0.18	14.64 ± 0.79	7.42 ± 0.21	4.48 ± 0.13	4.42 ± 0.16	2.55 ± 0.13	1.40 ± 0.12	5.82 ± 0.52	110.05
40	*Mentha* × *piperita* L. (peppermint)	Leaves	0.01 ± 0.03	11.14 ± 0.00	0.04 ± 0.00	0.16 ± 0.00	1.53 ± 0.01	2.44 ± 0.14	1.47 ± 0.08	1.05 ± 0.11	0.97 ± 0.00	1.23 ± 0.11	1.13 ± 0.07	31.41
41	*Moringa oleifera* Lam. (moringa tree)	Leaves	2.21 ± 0.37	32.42 ± 6.86	0.92 ± 0.21	7.82 ± 0.46	7.83 ± 1.08	19.02 ± 3.17	11.47 ± 1.91	10.93 ± 1.63	10.08 ± 1.98	11.69 ± 1.17	16.2 ± 1.06	266.90
42	*Musa acuminata* Colla (banana)	Peal	ND	0.42 ± 0.02	0.02 ± 0.00	0.08 ± 0.00	0.40 ± 0.01	0.48 ± 0.03	0.29 ± 0.02	0.10 ± 0.01	0.09 ± 0.01	0.02 ± 0.00	0.25 ± 0.20	4.55
43	*Musa acuminata* Colla (banana)	Fruit	ND	3.34 ± 0.37	0.01 ± 0.00	0.04 ± 0.00	0.41 ± 0.06	0.41 ± 0.04	0.25 ± 0.03	0.17 ± 0.02	0.05 ± 0.00	0.10 ± 0.02	0.07 ± 0.00	6.99
44	*Nigella sativa* L. (black cumin)	Seeds	0.69 ± 0.02	16.19 ± 1.49	0.43 ± 0.02	8.37 ± 1.11	2.66 ± 0.36	3.87 ± 0.43	2.33 ± 0.26	2.04 ± 0.13	2.58 ± 0.23	15.05 ± 1.02	6.30 ± 0.69	122.07
45	*Ocimum basilicum* L. (Mexican spice basil)	Leaves	0.12 ± 0.07	10.13 ± 0.00	0.12 ± 0.00	0.21 ± 0.00	9.61 ± 0.02	1.90 ± 0.18	1.15 ± 0.11	1.52 ± 0.06	0.84 ± 0.00	0.50 ± 0.07	2.37 ± 0.01	42.37
46	*Persea americana* Mill. (avocado)	Fruit	ND	2.63 ± 0.13	0.85 ± 0.16	2.74 ± 0.08	0.50 ± 0.01	2.93 ± 0.21	1.77 ± 0.13	1.66 ± 0.04	2.29 ± 0.05	0.20 ± 0.01	1.39 ± 0.40	60.92
47	*Phaseolus vulgaris* L. (common bean)	Seeds	0.09 ± 0.00	25.81 ± 0.28	0.81 ± 0.04	2.53 ± 0.03	23.92 ± 0.01	14.02 ± 0.05	8.46 ± 0.03	4.95 ± 0.02	4.30 ± 0.02	1.89 ± 0.05	4.92 ± 0.07	181.36
48	*Piper nigrum* L. (black pepper)	Seeds	0.01 ± 0.00	48.37 ± 1.75	0.28 ± 0.03	1.46 ± 0.12	0.24 ± 0.03	2.82 ± 0.35	1.70 ± 0.21	1.03 ± 0.03	0.99 ± 0.09	0.50 ± 0.11	1.15 ± 0.08	139.35
49	*Pisum sativum* var. *Abyssinicum* (A. Braun) Berger (decoco)	Seeds	0.63 ± 0.04	35.27 ± 7.84	1.39 ± 0.18	11.79 ± 2.38	2.58 ± 0.56	4.39 ± 0.65	2.65 ± 0.39	6.12 ± 0.34	3.62 ± 0.67	3.02 ± 0.22	7.17 ± 1.35	214.39
50	*Plectranthus edulis* (Vatke) Angew (Ethiopian potato)	Tubers	5.46 ± 0.73	27.13 ± 2.82	0.76 ± 0.20	3.90 ± 0.57	22.82 ± 1.96	5.74 ± 0.56	3.46 ± 0.34	4.46 ± 0.33	7.84 ± 0.83	2.27 ± 0.32	2.48 ± 0.25	153.29
51	*Pleurotus ostreatus* (Jacq. ex Fr.) P. Kumm. (oyster mushroom)	Fruit body	22.2 ± 9.95	15.69 ± 1.09	5.06 ± 0.68	3.64 ± 0.01	34.59 ± 5.33	28.69 ± 0.42	17.31 ± 0.25	22.63 ± 0.6	15.20 ± 0.13	8.56 ± 0.47	26.05 ± 3.86	400.01
52	*Ruta chalepensis* L. (rue)	Leaves	0.68 ± 0.23	27.25 ± 0.06	0.96 ± 0.38	30.10 ± 0.66	7.10 ± 0.37	7.73 ± 0.71	4.67 ± 0.43	6.70 ± 0.24	7.32 ± 0.62	8.03 ± 0.31	14.48 ± 0.93	215.83
53	*Rumex abyssinicus* Jacq. (mekmako)	Leaves	0.01 ± 0.00	2.45 ± 0.22	0.11 ± 0.01	0.97 ± 0.40	6.52 ± 0.49	3.58 ± 0.13	2.16 ± 0.08	1.84 ± 0.05	1.72 ± 0.07	0.60 ± 0.18	3.91 ± 0.26	47.13
54	*Salvia rosmarinus* Schleid. (rosemary)	Leaves	0.04 ± 0.01	28.13 ± 0.26	0.57 ± 0.08	0.71 ± 0.11	5.85 ± 0.92	2.28 ± 0.44	1.38 ± 0.27	1.00 ± 0.14	1.88 ± 0.15	0.84 ± 0.10	8.21 ± 0.06	80.97
55	*Sesamum indicum* L. (sesame)	Seeds	0.01 ± 0.01	7.67 ± 0.23	0.43 ± 0.01	0.74 ± 0.00	5.18 ± 0.01	2.30 ± 0.02	1.39 ± 0.01	0.95 ± 0.01	0.91 ± 0.01	0.74 ± 0.01	1.85 ± 0.02	44.31
56	*Solanum lycopersicum* L. (tomato)	Fruit	0.03 ± 0.01	18.74 ± 2.64	0.25 ± 0.02	0.38 ± 0.04	20.51 ± 3.10	3.29 ± 0.67	1.98 ± 0.40	1.43 ± 0.37	0.74 ± 0.08	1.12 ± 0.12	8.37 ± 1.64	137.15
57	*Syzygium aromaticum* (L.) Merr. & L.M.Perry (clove)	Flower	0.21 ± 0.00	18.69 ± 0.15	1.52 ± 0.00	5.19 ± 0.01	46.87 ± 0.00	6.78 ± 0.01	4.09 ± 0.01	6.05 ± 0.00	8.02 ± 0.01	4.83 ± 0.00	11.23 ± 0.02	261.19
58	*Thymus vulgaris* L. (thyme)	Leaves	0.01 ± 0.01	18.54 ± 1.59	1.16 ± 0.98	7.54 ± 2.58	12.51 ± 0.70	5.64 ± 1.13	3.40 ± 0.68	2.78 ± 0.33	3.28 ± 0.34	2.70 ± 0.22	4.96 ± 0.28	111.83
59	*Trigonella foenum-graecum* L. (fenugreek)	Seeds	0.03 ± 0.02	2.64 ± 0.14	0.70 ± 0.04	2.42 ± 0.04	0.58 ± 0.35	2.61 ± 0.10	1.57 ± 0.06	1.51 ± 0.05	2.15 ± 0.06	0.25 ± 0.04	1.11 ± 0.16	57.37
60	*Zingiber officinale* Roscoe (ginger)	Tubers	0.04 ± 0.01	21.05 ± 1.22	2.40 ± 0.29	6.95 ± 0.14	1.34 ± 0.16	9.88 ± 0.27	5.96 ± 0.16	4.97 ± 0.06	5.47 ± 0.31	2.00 ± 0.04	4.82 ± 0.32	248.50
Min			0.00	0.06	0.01	0.01	0.05	0.07	0.04	0.01	0.01	0.00	0.01	
Max			22.2	48.37	5.06	30.1	64.59	28.69	17.31	22.63	15.2	15.05	26.25	

*ND* not detected, *Total FAA* total free amino acid content

Oyster mushroom (*Pleurotus ostreatus* (Jacq. ex Fr.) P. Kumm.), a healthy food rich in protein, chitin, vitamins and minerals and which contains many bioactive molecules (Khan and Tania 2012), is ranked second among the important cultivated mushrooms in the world (Jose and Janardhanan 2000). In the present study, it had higher total FAA concentration (400.01 mg/g) as compared to all the tested samples (see Table 1) and the presence of such molecules may contribute to its characteristic flavor (Mau et al. 1998). The concentration of most of the FAAs was greater than 15.00 mg/g which is sufficient quantity for dermatological preparations for the management of dry skin condition. A total FAA content of 161.09 mg/g was reported by Kim et al (2009) after HPLC analysis on this type of mushroom originated from Korea and this was lower than the value obtained in the present study. The literature also reveals that there is a significant difference in the total FAA content of previously tested mushrooms (Sun et al. 2017) and this could be due to the difference in the harvesting time, growth condition, geographical origin in addition to the differences in extraction, derivatization, or quantification methods.

Being protein-rich substances, legume plants are expected to contain high amount of amino acids. Among the legume seeds included in the present study, dekoko (*Pisum sativum* var. *abyssinicum* (A. Braun) Berger), which obtains a premium price in local markets, contained relatively high amount of total FAAs followed by flaxseed (*Linum usitatissimum* L.), common bean (*Phaseolus vulgaris* L.), sunflower seeds (*Helianthus annuus* L.), soybean (*Glycine max* [L.] Merr.) (130.91–214.39 mg/g) (Table 1). The other legume seeds investigated were peanuts (*Arachis hypogaea* L.), fenugreek (*Trigonella foenum-graecum* L.) and sesame seed (*Sesamum indicum* L.) with a total FAA content of 73.62, 57.37 and 44.31 mg/g, respectively. The dominant FAAs in most of these plants were Arg, Asp, Glu, Asn, Gln, and Leu. Previous studies on some of these plants, namely flaxseeds (Panaite et al. 2017), common bean (Fukuji et al. 2019; Saboori-Robat et al. 2019), and sunflower seeds (Robinson 1975) also indicated that the stated FAAs are the dominant ones. However, the concentrations obtained were different may be due the difference in testing methodology and source of the materials. It is also mentioned in the literature that soybean seeds are able to store nitrogen mostly in the form of either proteins or FAAs (Takahashi et al. 2003).

Seeds of Ethiopian oat (*Avena abyssinica* Hochst.) and teff (*Eragrostis tef* (Zuccagni) Trotter) were selected and investigated from the cereal products. Ethiopian oat had very low total FAA concentration (4.41 mg/g) while teff had a total FAA content of 162.84 mg/g. The dominant FAAs in teff seeds were Glu, Asp, Asn, Ala, and Lys (11.39–26.61 mg/g).

The edible parts of 14 vegetables were included in the present study and the total FAA content was highest in broccoli (*Brassica oleracea* var *italica* Plenck) followed by garlic (*Allium sativum* L.), Ethiopian onion (*Allium spathaceum* Steud. ex A.Rich), cabbage (*Brassica oleracea* var. *capitata* (L.) Metzg*.*), Ethiopian mustard (*Brassica carinata*), black mustard (*Brassica nigra* L.), Ethiopian potato (*Plectranthus edulis* (Vatke) Agnew), tomato (*Solanum lycopersicum* L.), lettuce (*Lactuca sativa* L.) and carrot (*Daucus carota* subsp. *sativus* (Hoffm.) Schübl. & G. Martens) in decreasing order (60.25–291.90 mg/g on dry basis) (Table 1). Among the vegetables, relatively low amount of total FAA was obtained in cucumber (*Cucumis sativus* L.), pumpkin (Cucurbita maxima Duchesne), coriander (*Coriandrum Sativum*) and zucchini (*Cucurbita pepo* subsp. *pepo* convar. Giromontiina) (11.67–59.89 mg/g). Even though most of the FAAs were greater than 7.00 mg/g, the dominant FAAs found in broccoli and cabbage were Arg, Asp, and Glu. In a study by Gomes and Rosa (2001), 17 FAAs were detected in different broccoli cultivars and the dominant amino acids were Gln and Glu. A study by Oliveira (2008), reported the total FAA content ranged from 3.30 to 14.40 mg/g after HPLC/UV analysis, indicated that the dominant FAAs in cabbage were Arg, Pro, and Thr. Garlic and Ethiopian onion were dominated by FAAs such as Arg, Glu, Lys, Asn, Gln, Asp, Leu, Gln, Try, and Ala (Table 1). Another study by Lee and Harnly (2005) indicated that 18 FAAs were present in garlic and the dominant FAAs reported were Gln, Asn, Glu, Lys, Pro, and Ser. Ethiopian potato and tomato were also dominated with free form Gln, Asn, and Asp. Investigation on other potato species also indicated that FAAs of high concentration were obtained after extracting with hot water (Furudate and Meguro 2001). Even though it is mentioned in the literature that Cit occurs most abundantly in Cucurbitaceous plants (Joshi and Fernie 2017), zucchini and cucumber had low concentration of this compound (Table 1).

Among the fruits and fruit peals included in this study, avocado (*Persea americana* Mill.) had the highest total FAA content (60.92 mg/g), followed by pineapple (*Ananas comosus* (L.) Merr), watermelon (*Citrullus lanatus* (Thunb.) Matsum. & Nakai), papaya (*Carica papaya* L.), strawberry (*Fragaria* × *ananassa* DUCHESNE), banana (*Musa acuminata* Colla), and mango (*Mangifera indica* L.) with a total FAA contents in the range of 1.72–54.68 mg/g. The peals of banana and mango were also tested but their FAA contents were very low (total FAA concentrations of 4.55 and 0.86 mg/g, respectively). The dominant FAAs in most of the fruit samples were Asn, GABA, Asp, Glu, Gln, Ala, Ser, and Lys. A study by Zeng et al (2015) on different fruit juices, FAAs such as Asp, Glu, Asn, Ser, and Gln were the dominant ones in most samples which shows almost the same pattern as the present study.

Spices commonly used in Ethiopian traditional dishes have also promising amount of FAAs (Table 1). Ginger (*Zingiber officinale* Roscoe), a plant with diverse biological activities (Zhao et al. 2011), had total FAA content of 248.50 mg/g. The dominant FAAs were Asp, Asn, Ala, Glu and Arg (9.88–83.40 mg/g). Met was found at a very low concentration (0.04 mg/g). Ogbuewu et al. (2014) who tested 17 amino acids reported a total amino acid content of 256.10 mg/g, also indicated Arg as the most dominant amino acid and Met at relatively lower concentration compared to the other FAAs. Cumin (*Cuminum cyminum* L.) and black cumin (*Nigella sativa* L.), which are active reservoirs of numerous bioactive compounds with various therapeutic applications, had total FAA concentrations of 164.20 and 122.07 mg/g, respectively. FAAs such as Arg, Asn, Asp, Glu, Pro, and Ala were the most abundant FAAs in both of them. Reports on cumin also indicate that the seeds of this plant are rich in FAAs (Badr and Georgiev 1990; Toghrol and Daneshpejouh 1974). Moreover, a study by Yimer et al. (2019) also revealed that Glu, Arg, and Asp are the major amino acids found in black cumin which is consistent with the present study. Hot peppers (*Capsicum annuum* L.) and black pepper (*Piper nigrum* L.) contribute a major share in the Ethiopian spice scenario. The total FAA contents of these plants were 205.68 and 139.35 mg/g, respectively. Free forms of Asn, Ala, GABA, and Asp were found at high concentrations (6.94–54.06 mg/g) in both plants. A study on hot pepper seeds grown in China, 18 amino acids were detected and the main amino acids were Glu and Asp (both of which had concentrations of above 20.00 mg/g) (Zou et al. 2015). The remaining spices, namely, rosemary (*Salvia rosmarinus SCHLEID*.), lemon grass (*Cymbopogon citratus* (DC.) Stapf) and cinnamon (*Cinnamomum verum* J. Presl) had a total FAA contents of less than 81.00 mg/g.

The leaves of some herbs, namely, chamomile (*Matricaria chamomilla* L.), peppermint (*Mentha* × *piperita* L.), Mexican spice basil (*Ocimum basilicum* L.), rue (*Ruta chalepensis* L.), and thyme (*Thymus vulgaris* L. were also included in the present study. Highest total FAAs were found in rue, chamomile and thyme (110.05–215.83 mg/g) (Table 1). Chamomile, which is often referred to as the “star among medicinal species”, is a well-known medicinal plant species from the Asteraceae family (Singh et al. 2011). The present study indicated that this plant is dominated with FAAs such as Asn, Gln, Asp, Glu, Lys, Arg, and Pro. A study by Ma et al. (2015), 15 FAAs were detected and analyzed by HPLC and the dominant FAAs reported were Pro, Ala, Ser, Glu, Asp, and Arg. Mekmeko (*Rumex abyssinicus* Jacq.), another perennial herb, had a total FAA content of 47.10 mg/g.

Among the seven indigenous aloe species included in this study, relatively high total FAA content was obtained in *Aloe percrassa* Tod., *A. tewoldei* M.G. Gilbert & Sebsebe and *A. ankoberensis* M.G. Gilbert & Sebsebe (73.06–76.70 mg/g). The dominant FAAs were slightly different in the different species of aloe. In previous study by Kim et al. (2013), a total of 24, 23 and 17 amino acids were detected in three species of aloe, namely, *A. vera* (L.) Burm.f., *A. saponaria* (Aiton) Haw., and *A. arborescens* Mill., respectively, indicating that different species can have different composition.

The leaves of other plants grown in Ethiopia were also included. Moringa (*Moringa oleifera* Lam.), a highly valued plant with exceptional nutritional value and array of health benefits, is widely grown in many tropical and subtropical countries. In the present study, the total FAA content of the leaf part of moringa tree was 449.71 mg/g. The dominant amino acids were Asg, Ala, Asp, Glu, Ser, and Leu. Hemp (*Cannabis sativa* L.) and clove (*Syzygium aromaticum* (L.) Merr. & L.M. Perry)) had total FAA concentration of 195.01 and 261.29 mg/g, respectively. FAAs such as Asp, Gln, Asn, Glu, Lys, and Tyr were the dominant ones in both. Previous studies on protein isolates of hemp also revealed that the plant had high amount of amino acids (Tang et al. 2006).

In general, the present study revealed that FAAs for cosmeceutical applications can be obtained from several nature-based resources. As reported by Hussain et al (2019), the concentration of each FAA in the stratum corneum of healthy human skin is less than 10.00 nmol/mg indicating that only very low amount of these bioactive molecules are required in formulations pertaining to replacement therapies for the treatment of dry skin conditions. As can be seen from the results in Table 1, natural resources including but not limited to oyster mushroom, broccoli, garlic, Ethiopian onion, ginger, cabbage, cumin, and decoco contain a significant amount of most of the FAAs. Among the tested FAAs, free Asp, Asn, Glu, Arg, Gln, GABA, His, Ala, Ser, Thr, Tyr, Try, Leu, Ile, Val, Pro, Phe, Gly, Pro, and Lys were dominantly found in these and the other plants. Even though the remaining FAAs, namely, Tau, Cit, Orn, oxyproline, *O*-acetylserine, and methionine oxide were found at low concentrations in most of the tested samples, they were found in sufficient quantity in some of the plants and in the mushroom. For example, oyster mushroom can be considered as source of Met, Cys and Orn. Moreover, sufficient amount of Tau and *O*-acetylserine were obtained in black mustard; and reasonable content of Cit was obtained in garlic. Hence, a crude extract of some of the stated plants have all the required FAAs and use of such resources for cosmeceutical applications has a great advantage over other sources (synthetic, enzymatic, and fermentation) in terms of economic benefits besides other nature-related advantages.

The natural products that had the highest amount of FAAs as per the current study have also been used in many cosmeceutical preparations. For example “Birch Water Purifying Essence”, “Larecea™ Extract”. and “Onion Juice Cream” are some examples of commercially available cosmeceutical preparations which contain extracts from mushroom, broccoli, and onion as their main components. Despite the few commercial moisturizers that contain nature-based extracts, to our knowledge, much research work has not been done on topical formulations loaded with plant or mushroom-based FAAs. The current study gives a clear insight to the natural products with high amounts of FAAs and will help to fill the research gap in topical preparations loaded with plant or mushroom-based FAA.

## Conclusion

In the present study, the FAA content of selected Ethiopian plant and mushroom species were investigated. The results revealed that significant amount of all the FAAs found in NMF were obtained in most of the investigated materials. Among the tested species, those with very short harvesting period (such as oyster mushroom, broccoli, garlic, ginger, cabbage, and pepper) can be considered as sources of these bioactive molecules. Simple extraction of FAA from natural resources for cosmeceutical preparation can be considered as alternative method to synthetic, enzymatic and fermentation methods, especially in low-income countries. In addition to its therapeutic advantages, use of such nature-based compounds for cosmeceutical purpose in biodiversity-rich countries like Ethiopia could have economic benefit. It also helps in fostering modern natural remedial approaches and fulfills customer satisfactions.

## Supplementary Information

Below is the link to the electronic supplementary material.

Supplementary file1 (XLSX 53 KB)

## Data Availability

The original data obtained from the calibration curves (in nmol/mg) are attached in the electronic supplement.
